# Supplementary material for: Multilevel modeling of county-level excessive alcohol use, rurality, and COVID-19 case fatality rates in the US
Source: PLoS One. 2021 Jun 17;16(6):e0253466. doi: 10.1371/journal.pone.0253466 (PMC8211222; doi:10.1371/journal.pone.0253466)
Supplement: Attachment — Submitted filename: Comments_PLOSone_03.24.2021.pdf [file pone.0253466.s001.pdf]

This paper explored the relationship between county-level excessive alcohol consumption, rurality and COVID-19 case-fatality rate using multilevel beta regression analyses. The authors make a compelling argument for the pathway through which a higher prevalence of excessive alcohol consumption may affect COVID-19 fatality. They also make a compelling argument for the reversed associations between the outcome and focal predictor. The paper is also methodologically sound. However, as indicated in the feedback below, some of the discussion and conclusion points are in contrast to the results shown in the tables. Minor revisions are suggested.

Tables.

Table 1. For ease of readability, the authors should consider providing summary statistics in table 1 for only the covariates (second part of table 1) and having the first part showing case-fatality rates for values of the focal predictor as a separate table.

Table 3. The low alcohol mid rurality coefficient as 0.00, the coefficient should be rounded to a non-zero value. Same applies to “percentage of a county’s adult population with a BMI of 30 or higher” covariate and several standard errors.

Discussion.

Page 14 line 4-6 “We found that the burden of higher case fatality rates lied predominately in mid alcohol use/mid rural and high alcohol use/high rural counties.” However, table 3 shows that the mid alcohol /mid rural was not statistically significant and also did not have the largest effect size (the mid alcohol/high rural and high alcohol/mid rural were significant).

Page 15 line 2 “Given indications that alcohol use is associated with higher COVID-19 mortality in some areas,…” this is in contrast to the index study finding and the authors do not provide any citation to support the statement.

Page 15 line 21-page 16 line 1 “our findings demonstrate disparate COVID-19 fatality burdens in some counties, in particular counties with mid/high excessive alcohol use and counties with mid/high levels of rurality.” This again is not supported by the study findings. Table 1 and table 2 indicate that the case fatality burden is more for the low alcohol/low rural counties.
